# Dysregulated Immune Responses by ASK1 Deficiency Alter Epithelial Progenitor Cell Fate and Accelerate Metaplasia Development during *H. pylori* Infection

**DOI:** 10.3390/microorganisms8121995

**Published:** 2020-12-14

**Authors:** Yoku Hayakawa, Yoshihiro Hirata, Masahiro Hata, Mayo Tsuboi, Yukiko Oya, Ken Kurokawa, Sohei Abe, Junya Arai, Nobumi Suzuki, Hayato Nakagawa, Hiroaki Fujiwara, Keisuke Tateishi, Shin Maeda, Kazuhiko Koike

**Affiliations:** 1 Department of Gastroenterology, Graduate School of Medicine, The University of Tokyo, 7-3-1 Hongo, Bunkyo-ku, Tokyo 113-8655, Japan; 2 Division of Advanced Genome Medicine, The Institute of Medical Science, The University of Tokyo, Tokyo 108-8639, Japan; 3 Department of Gastroenterology, The Institute for Adult Diseases, Asahi-Life Foundation, Tokyo 103-0002, Japan; 4 Department of Gastroenterology, Yokohama City University 3-9 Fukuura, Kanazawa-ku, Yokohama 236-0004, Japan

**Keywords:** *Helicobacter pylori*, ASK1, gastritis, metaplasia, progenitors

## Abstract

The mechanism of *H. pylori*-induced atrophy and metaplasia has not been fully understood. Here, we demonstrate the novel role of Apoptosis signal-regulating kinase 1 (ASK1) and downstream MAPKs as a regulator of host immune responses and epithelial maintenance against *H. pylori* infection. ASK1 gene deficiency resulted in enhanced inflammation with numerous inflammatory cells including Gr-1+CD11b+ myeloid-derived suppressor cells (MDSCs) recruited into the infected stomach. Increase of IL-1β release from apoptotic macrophages and enhancement of TH1-polarized immune responses caused STAT1 and NF-κB activation in epithelial cells in ASK1 knockout mice. Dysregulated immune and epithelial activation in ASK1 knockout mice led to dramatic expansion of gastric progenitor cells and massive metaplasia development. Bone marrow transplantation experiments revealed that ASK1 in inflammatory cells is critical for inducing immune disorder and metaplastic changes in epithelium, while ASK1 in epithelial cells regulates cell proliferation in stem/progenitor zone without changes in inflammation and differentiation. These results suggest that *H. pylori*-induced immune cells may regulate epithelial homeostasis and cell fate as an inflammatory niche via ASK1 signaling.

## 1. Introduction

*Helicobacter pylori* (*H. pylori*) is associated with various human gastric diseases, including gastric ulcer, chronic gastritis, and gastric neoplasia [1,2]. During *H. pylori*-induced chronic gastritis, the initial pathological change is oxyntic atrophy or loss of parietal and chief cells [3]. Parietal and chief cell loss is considered to alter the status of proliferation or differentiation in oxyntic glands, and lead to induce metaplastic changes [4,5,6,7]. Recent published papers have identified several stem cell markers in the stomach [8,9,10,11,12,13,14]. However, it has been poorly understood how gastric stem/progenitor cells could differentiate to metaplasia or dysplasia during *H. pylori* infection.

In humans and mice, the *H. pylori*-infected gastric mucosa exhibits high levels of proinflammatory cytokines including IL-8, IL-1β, TNF-ɑ, and IL-6 [15,16,17,18,19,20]. Especially in mice, TH1-polarized cellular immune responses are highly involved, resulting in elevated levels of IFN-γ, IL-12, and IL-18 [17,21,22,23]. These numerous cytokines are produced by various kinds of recruited inflammatory cells such as macrophages, neutrophils, lymphocytes, dendritic cells, or fibroblasts, and such inappropriate immune responses in the host may eventually cause the gastric phenotype by *H. pylori* [23,24,25,26]. Indeed, polymorphisms in proinflammatory cytokine genes have been proved to increase the risk of gastric cancer [27,28].

A series of in vitro reports have shown the importance of *cag*-PAI in the pathogenesis of *H. pylori*-induced gastric epithelial changes. CagA that infected into host cells leads to the phosphorylation of CagA by host cell kinases, resulting in activation of SHP-2 tyrosine phosphatase, NF-κB signaling pathways, and mitogen-activated protein kinase (MAPK) signaling pathways [29,30,31,32,33]. Apoptosis signal-regulating kinase 1 (ASK1) is a ubiquitously expressed MAPK kinase kinase (MAP3K), which activates the c-Jun N-terminal kinase (JNK) and p38 signaling pathways and is required for both oxidative stress and cytokine-induced apoptosis [34]. Previously, our studies have shown that ASK1 has important functions in gastric epithelial cells for controlling proliferation and cell cycle both in vivo and in vitro [35,36]. It was also reported that ASK1 and downstream signaling play a critical role in various cancer development, including colon, pancreas, and liver cancers [37,38,39].

It has been also reported that ASK1 is highly involved in immune responses. Lipopolysaccharide (LPS) or tumor necrosis factor-ɑ (TNF-ɑ) induced the formation of a complex of TRAF6 and ASK1, and subsequent activation of the ASK1-p38 pathway in inflammatory cells [40,41]. During colonic inflammation, ASK1 regulates bacterial killing ability in macrophages and controls their cell fate, which finally affects systemic immune responses and carcinogenesis in mice [37]. However, it has not been fully elucidated whether ASK1 plays a role in *H. pylori*-induced host immune responses and affects epithelial differentiation.

In this study, we demonstrate that lack of ASK1 causes severely enhanced mucosal changes in the *H. pylori*-infected stomach. In particular, ASK1 in inflammatory cells plays a critical role for preventing gastric atrophy and metaplastic changes through IL-1β release, TH1-polarized immune responses, and the recruitment of Gr-1+CD11b+ myeloid-derived suppressor cells (MDSCs), while ASK1 in epithelial cells regulates stem/progenitor cell proliferation. Moreover, abnormal inflammatory responses that were caused by ASK1 deficiency resulted in altered distribution of gastric facultative progenitor cells in metaplastic lesions. These results highlight the novel role of ASK1 in the interaction between host immune responses and epithelial homeostasis during *H. pylori* infection.

## 2. Materials and Methods

### 2.1. Mice and Helicobacter Infection Model

The generation of *ASK1^–/–^* and *ASK2^–/–^* mice was described previously [42,43]. *ASK1^–/–^* and *ASK2^−/−^* mice were back-crossed into the C57BL/6 strain at least 18 times. C57BL/6 *WT* mice were purchased from Clea Japan (Tokyo, Japan). The *Helicobacter* strain used in this study was the *H. pylori* Sydney strain 1 (SS-1) [44] and PMSS-1, which was kindly provided from Dr. Anne Muller [45]. *H*. *pylori* was grown as described previously [46]. Six-week-old male *WT* and *ASK1^−/−^* mice were inoculated against *H*. *pylori* using three oral gavage dosages per week (10^8^ colony-forming units/0.2 mL). The stomachs were removed and used for histological and immunoblot analyses. For quantitative assessment of *H. pylori* colonization, one section of each stomach was transferred to a tube containing *Brucella* broth (Becton, Dickinson and Company, Sparks, MD, USA), and homogenized. Serial dilutions were plated on *Brucella* agar (Becton, Dickinson and Company) plates to determine bacterial loads. All of the experimental protocols (P19-102) were approved by the Ethics Committee for Animal Experimentation and conducted in accordance with the Guidelines for the Care and Use of Laboratory Animals of the Graduate School of Medicine, the University of Tokyo, and the Institute for Adult Diseases, Asahi Life Foundation, Tokyo, Japan. 

### 2.2. Reagents

Anti-phospho-JNK, anti-JNK, anti-phospho-p38, anti-p38, anti-phospho-MKK3, anti-phospho-MKK4, anti-phospho-ATF2, anti-phospho-MK2, anti-phospho-CREB, anti-phospho-cjun, anti-phospho-IκBɑ, anti-phospho-STAT1, anti-phospho-STAT3, anti-IL-1β, anti-cleaved caspase-3, and anti-phospho-ASK1 antibodies were purchased from Cell Signaling Technology (Danvers, MA, USA). Anti-ASK1 antibody was provided from Dr. Hidenori Ichijo. Anti-F4/80 antibody was obtained from Serotec. Anti-CD4, anti-caspase-1, and anti-Dclk1 antibodies were purchased from Abcam (Cambridge, MA, USA). Anti-TFF2 antibody was kindly provided by Dr. Sachiyo Nomura. Anti-proton pump and anti-Cdx2 antibodies were obtained from Santa Cruz (Santa Cruz, CA, USA). Anti-BrdU antibody was obtained from Dako (Santa Clara, CA, USA). Anti-actin antibody was purchased from Sigma. Anti-cyclin D1(AB3) was acquired from Invitrogen (Waltham, MA, USA). 

### 2.3. Bone Marrow Chimeric Mice Generation

Bone marrow transplantation was performed as described previously [37]. Cell suspensions from female *WT* or *ASK1^−/−^* bone marrow were prepared from femurs and tibias, filtered, and counted. Male recipient mice received a single intravenous injection of 1 × 10^7^ bone marrow cells, after being irradiated with 9.5 Gy x-rays. For 4 weeks following the transplant, drinking water was replaced with water containing neomycin sulfate/polymyxin B sulfate mixture. Transplanted mice were treated with *H. pylori* 6 weeks after irradiation. Genomic DNA and protein were extracted from spleen, and bone marrow chimerism was determined by PCR and Western blotting.

### 2.4. Cell Lines 

AGS cell line was purchased from ATCC. The cell lines were cultured in Ham’s F-12 medium supplemented with 10% fetal bovine serum. 

### 2.5. Immunostaining 

Tissues were fixed in 10% formaldehyde, dehydrated, embedded in paraffin, and sectioned as described previously [47]. The sections were deparaffinized and incubated overnight at 4 °C with indicated antibodies. Binding of the primary antibody was detected with anti-rabbit IgG (Vector Laboratories), followed by visualization with 3,3’-diaminobenzidine (Sigma-Aldrich). To assess cellular proliferation, mice were injected i.p. with 120 mg/kg of bromodeoxyuridine (BrdU; BD-Pharmingen, San Diego, CA, USA) 90 min before sacrifice, and gastric tissues were stained with anti-BrdU antibody. For immunofluorescence, the paraffin-embedded or frozen sections were incubated with primary antibodies, followed by secondary Alexa555 or Alexa488 IgG antibody (Invitrogen). TUNEL was analyzed by using the Apoalert DNA Fragmentation Assay kit (Takara, Shiga, Japan).

### 2.6. Western Blotting, Immunoprecipitation, and ELISA

Protein lysates were prepared from cells or tissues, separated by sodium dodecyl sulfate-polyacrylamide gel electrophoresis (SDS-PAGE), transferred to polyvinylidene difluoride membranes (Millipore, St. Louis, MO, USA). The membrane was probed with primary antibodies, and then incubated with the secondary antibody. Immunocomplexes were detected using the enhanced chemiluminescence system (Amersham Biosciences, Buckinghamshire, UK). For immunoprecipitation, samples were lysed in radioimmunoprecipitation assay buffer and immunoprecipitated with 50 μL of protein A/G Sepharose beads (Santa Cruz Biotechnology) overnight at 4 °C using the indicated antibodies. The beads were washed 3 times with radioimmunoprecipitation buffer and then analyzed by SDS-PAGE. Cytokine levels were measured using ELISAs (R&D Systems, Minneapolis, MN, USA).

### 2.7. RNA Analysis

Total RNA was extracted from the tissues using the Nucleospin RNA II Kit (Takara). The first-strand complementary DNA was synthesized using the ImProm-II Reverse Transcription System (Promega, Madison, WI, USA). Amplification was performed using the ABI PRISM 7000 Quantitative PCR System (Applied Biosystems). The different mRNAs were quantitated by Real-Time PCR using *Gapdh* mRNA for normalization. The primer sequences used are available upon request.

### 2.8. Statistical Analyses 

Differences between means were compared using Student’s *t*-test. *P* values < 0.05 were considered statistically significant.

## 3. Results

### 3.1. Loss of ASK1 Exacerbated Atrophic and Metaplastic Changes in H. pylori-Infected Stomach

**Figure 1:**
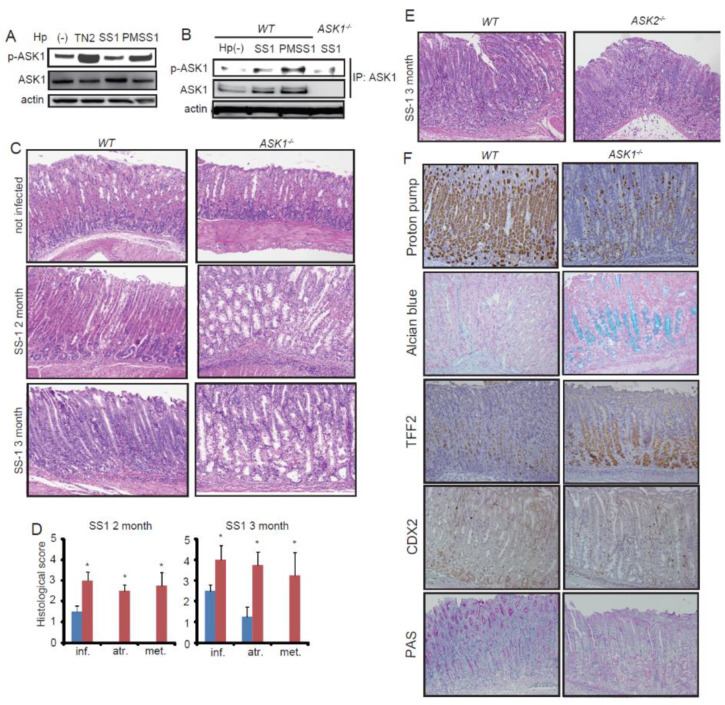
Loss of ASK1 exacerbated atrophic and metaplastic changes in *H. pylori*-infected stomach. (**A**) ASK1 phosphorylation in AGS cells that were transfected with ASK1-overexpressing vector. Cells were harvested 24 h after *H. pylori* infection. (**B**) ASK1 phosphorylation in mouse stomachs from control (Hp(-)), SS-1-infected, and PMSS-1-infected mice (3 mo post-infection). (**C**) Representative H&E staining of *WT* and *ASK1^−/−^* mouse stomach with/without *H. pylori* SS-1 infection. Original magnification, ×100. (**D**) Histological scoring of SS-1-infected *WT* and *ASK1^−/−^* mouse stomach. Inf., inflammation; atr., atrophy; met., metaplasia; hyp., hyperplasia. Data are shown as mean ± SD. * *p* <0.05 compared to infected *WT* mice. (**E**) H&E staining of SS-1-infected *WT* and *ASK2^−/−^* mouse stomach. Mice were sacrificed 3 mo after SS-1 infection. Original magnification, ×100. (**F**) Immunohistochemical staining of SS-1-infected *WT* and *ASK1^−/−^* mouse stomachs. Original magnification, ×100.

Type IV secretion system (TFSS) has been reported to be important for MAPK pathway activation in gastric epithelial cells [29,30,31,32]. Although *H. pylori* SS-1 is a well-known strain which can colonize to mouse stomach, functional TFSS is disrupted in this strain [48]. PMSS-1 strain, which was the primarily isolated strain from SS-1-infected patients, has efficient TFSS, and induces more aggressive gastritis in mouse stomach than SS-1 [19,45], suggesting the possibility that efficient TFSS could cause more severe inflammation by activating epithelial MAPK signaling cascade. Thus, first we used both SS-1 and PMSS-1 strains in order to analyze ASK1 activation in vitro and in vivo. In AGS cells, *H. pylori* TN2 strain and PMSS-1 could increase the phosphorylated level of ASK1, while SS-1 did not, suggesting that TFSS is necessary for ASK1 activation in gastric epithelial cells (Figure 1A). Next, we infected *WT* and *ASK1^−/−^* mice with SS-1 and PMSS-1 for up to 3 months and examined protein expression in the stomachs. Both infected stomachs showed increased level of ASK1 phosphorylation compared to noninfected stomach, and PMSS-1-infected stomach displayed greater level of phosphorylated ASK1 than SS-1-infected stomach (Figure 1B). These results indicate that PMSS-1 activates ASK1 in gastric epithelium via phosphorylation more strongly than SS-1.

Noninfected *WT* and *ASK1^−/−^* mice did not show significant differences in the morphology of their stomachs (Figure 1C) as reported previously [35]. However, *ASK1^−/−^* mice infected with SS-1 exhibited dramatically increased level of inflammation, gastric atrophy, and metaplasia, compared to *WT* infected mice after 2 and 3 months (mo) infection (Figure 1C,D). PMSS-1 induced more severe inflammation and atrophic changes in *WT* mice than SS-1 as reported previously [45]; however, ASK1 deficiency further exacerbated gastric atrophy and metaplasia even in the setting of PMSS-1 infection (Figure S1A,B). There were no significant differences between *WT* and *ASK1^−/−^* mice in SS-1 and PMSS-1 colonization (Figure S1C). These results suggest that systemic ASK1 deficiency enhanced inflammation and metaplastic changes independently from TFSS-mediated signaling and bacterial clearance. 

It has been reported that ASK2 is closely related to ASK1 but can activate the JNK and p38 pathways only by forming a heteromeric complex with ASK1, and thus *ASK1^−/−^* mice lack ASK2 function [43]. To determine whether the more prominent phenotype in *ASK1^−/−^* mice was dependent on ASK1 or ASK2 function, we infected *WT* and *ASK2^−/−^* mice with *H. pylori*. After 2 mo infection, infected *ASK2^−/−^* mice did not show enhanced mucosal changes compared to *WT* mice (Figure 1E). 

Next, we performed immunohistochemistry (IHC) of SS-1-infected *WT* and *ASK1^−/−^* stomach. Alcian blue and proton pump staining confirmed that the development of metaplasia and parietal cell loss was significantly enhanced in *ASK1^−/−^* stomach (Figure 1F). It has been reported that there are two types of metaplasia in the stomach: one is the traditional intestinal metaplasia, which is characterized by goblet cell morphology and Cdx2-positive; the other is spasmolytic polypeptide expressing metaplastic lineage (SPEM), a metaplasia in the gastric fundus resembling deep antral gland cells and expressing Trefoil Factor 2 (TFF2) [49,50,51,52]. The metaplastic lesion found in *ASK1^−/−^* stomach was TFF2-positive and CDX2-negative, suggesting that these metaplastic changes were SPEM (Figure 1F). Interestingly, although pit-cell hyperplasia is generally accompanied with atrophy and metaplasia during *Helicobacter* infection, PAS staining showed the decrease of pit-cell hyperplasia in *ASK1^−/−^* stomach compared to *WT* stomach, in contrast to atrophy and metaplasia (Figure 1F). These results indicate that ASK1, not ASK2, is a critical regulator of the development of inflammation and metaplasia in the stomach.

### 3.2. ASK1 Regulates Stem/Progenitor Cell Proliferation Through Downstream MAPK Activation

**Figure 2:**
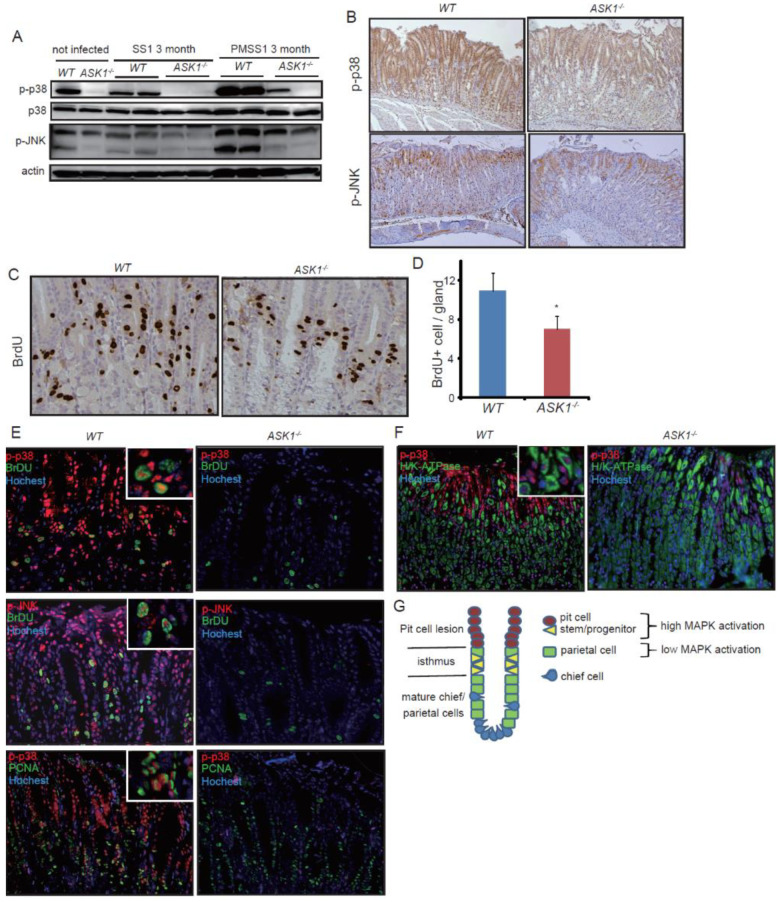
ASK1 regulates stem/progenitor cell proliferation through downstream MAPK activation. (**A**) Immunoblotting of *WT* and *ASK1^−/−^* mice stomach with/without *H. pylori* infection with the indicated proteins. (**B**) Immunohistochemical staining of SS-1-infected *WT* and *ASK1^−/−^* mouse stomachs with the indicated proteins. Original magnification, ×100. (**C**,**D**) BrdU staining (**C**) and the numbers of BrdU+ cells per gland (**D**) in SS-1-infected *WT* and *ASK1^−/−^* mice. Original magnification, ×200. Data are shown as mean ± SE. * *p* < 0.05 compared to infected *WT* mice. (**E**,**F**) Immunofluorescence in SS-1-infected *WT* and *ASK1^−/−^* mice. Original magnification, ×200. Colors of the stained proteins are indicated in the panels. (**G**) Schema of corpus gland structure.

We next tested phosphorylation of JNK and p38, the downstream molecules of ASK1. PMSS-1 enhanced phosphorylation of both JNK and p38 in *WT* mice, while the effect of SS-1 infection was minimal. Interestingly, *ASK1^−/−^* mice showed markedly decreased levels of MAPK activity either with or without *Hp* infection, compared to *WT* mice (Figure 2A). In contrast, immunoblotting of *ASK2^−/−^* stomach and spleen showed same levels of phosphorylated p38 (p-p38) and phosphorylated JNK (p-JNK) between *WT* and *ASK2^−/−^* mice (Figure S2A). Consistent with the immunoblotting results, immunostaining revealed that *ASK1^−/−^* mice showed decreased expression of phopho-p38 and phospho-JNK in the nuclei, which was most evident in the isthmus-surface area of gastric mucosa (Figure 2B). Total p38 expression was preserved in the cytoplasm in *WT* and *ASK1^−/−^* stomach (Figure S2B). These results suggest that epithelial MAPK activation is mostly restricted to the isthmus and pit cell region and largely regulated by upstream ASK1 in the stomach.

To investigate the effect of ASK1 on gastric epithelium more precisely, we next performed BrdU labeling assay. In the glandular stomach, BrdU-positive cells were seen in the isthmus region where stem/progenitor cells are considered to reside (Figure 2C). BrdU staining showed the significantly decreased numbers of BrdU+ cells in *ASK1^−/−^* stomach compared to *WT* stomach (Figure 2D). Double immunofluorescence staining revealed that the phosphorylation of p38 and JNK was found to be strongest in the isthmus and surface pit region, and that approximately 50% of BrDU-positive actively cycling stem/progenitor cells was positive for phospho-p38 and phospho-JNK (Figure 2E). In contrast, *ASK1^−/−^* stomach showed quite weak or no JNK/p38 activation in the isthmus and pit cells (Figure 2E). Proliferating cell nuclear antigen (PCNA) staining demonstrated similar results to BrdU staining (Figure 2E). We observed weak p38 phosphorylation in mature parietal cells in *WT* mice, but p38 phosphorylation in *ASK1^−/−^* parietal cells was almost absent (Figure 2F). These suggest that p38 and JNK are highly activated in the isthmus stem/progenitor cells and the surface pit cells, and activation of p38 and JNK may promote isthmus proliferation and pit-cell differentiation (Figure 2G).

### 3.3. ASK1 Deficiency Enhanced NF-κB and STAT1 Activation in the Hp Infected Stomach

**Figure 3:**
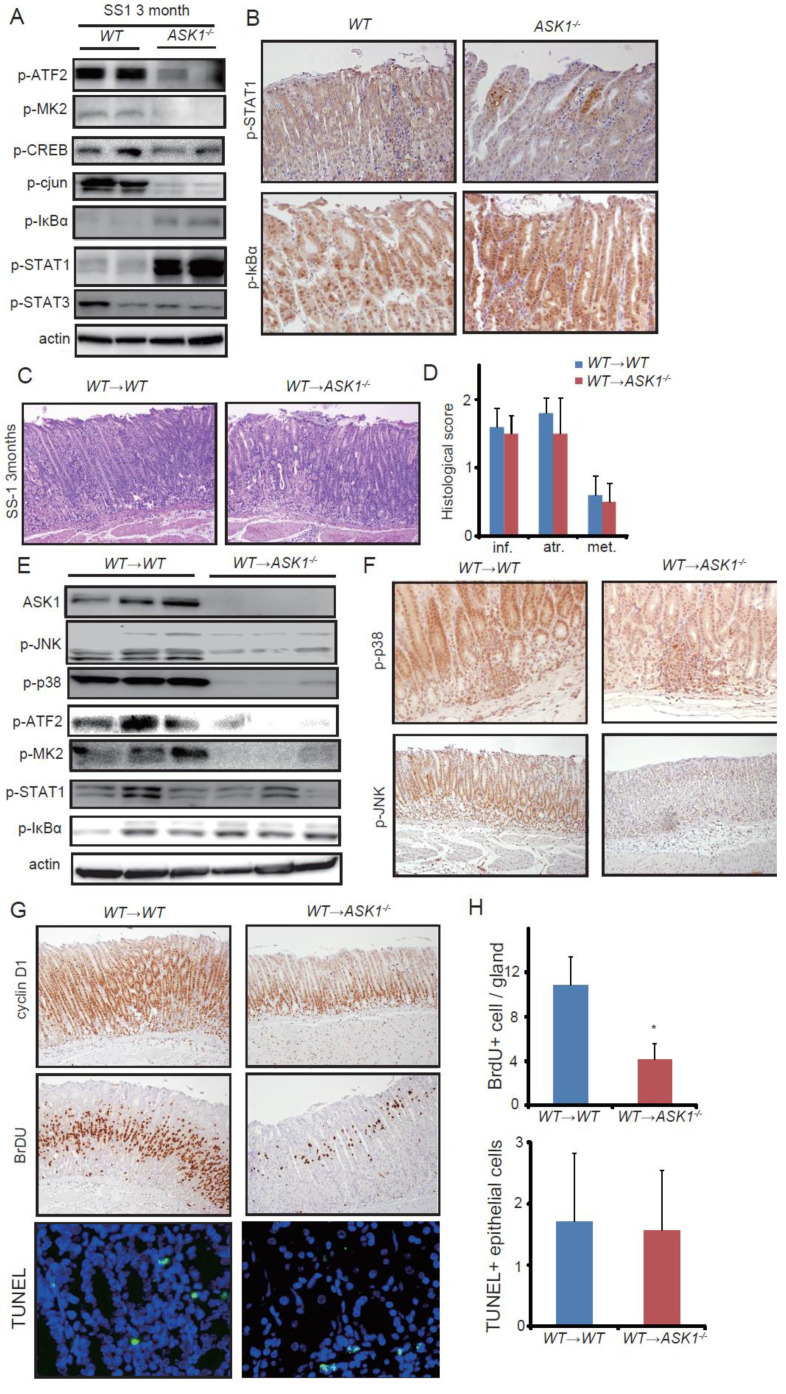
Epithelial ASK1 does not contribute to inflammation and metaplasia development, but mediates proliferation. (**A**) Immunoblotting of the indicated proteins in SS-1-infected *WT* and *ASK1^−/−^* mouse stomachs. (**B**) Immunohistochemical staining of SS-1-infected *WT* and *ASK1^−/−^* mouse stomachs with the indicated proteins. (**C**) H&E staining of *WT* (*WT*→*WT*) and *ASK1^−/−^* (*WT*→*ASK1^−/−^*) mice transplanted with *WT* bone marrow. Mice were sacrificed 3 mo after SS-1 infection. Original magnification, ×100. (**D**,**E**) Histological scoring (**D**), and immunoblotting (**E**) of SS-1-infected *WT*→*WT* and *WT*→*ASK1^−/−^* mice. (**F**–**H**) Immunohistochemistry of the indicated proteins (**F**,**G**) and the numbers of BrdU and TUNEL positive cells per gland (**H**) in SS-1-infected *WT*→*WT* and *WT*→*ASK1^−/−^* mice. Original magnification: TUNEL, ×400; p-p38, ×200; p-JNK, cyclin D1, and BrdU, ×100. Data are shown as mean ± SE. * *p* < 0.05 compared to infected *WT*→*WT* mice.

To further address downstream transcriptional factors that are affected by ASK1 in *Hp-*infected epithelium, we performed additional immunoblotting. It is known that JNK activates c-jun by phosphorylation and that p38 regulates the activation of several transcriptional factors, including activating transcription factor (ATF), MAPK-activated protein kinase 2 (MK2), and cAMP response element binding protein (CREB) [53,54,55]. Immunoblotting of SS-1 and PMSS-1 infected stomachs revealed the decrease levels of phopho-ATF, phospho-MK2, and phospho-c-jun, but did not show the deference in phospho-CREB (Figure 3A and Figure S3). MAPK pathways have various interactions with other signaling pathways such as NF-κB and signal transducer and activator of transcription family (STAT) signaling, and these signaling pathways are also important for gastric inflammation and carcinogenesis [56,57,58,59]. Interestingly, infected *ASK1^−/−^* stomach showed enhanced activation of NF-κB and STAT1, but not STAT3 (Figure 3A and Figure S3). In immunohistochemistry, we observed stronger activation of NF-κB and STAT1 in epithelial cells in infected *ASK1^−/−^* mice compared to infected *WT* mice (Figure 3B). These results suggest that downregulation of p38 and JNK in *ASK1^−/−^* stomach might induce compensatory activation of NF-κB and STAT1 pathways, which potentially lead to gastric atrophy and metaplasia.

### 3.4. Epithelial ASK1 Did Not Affect Inflammatory and Metaplastic Changes After H. pylori Infection

In order to explore the precise function of ASK1 in these phenotypes, we next transplanted bone marrow cells derived from *WT* mice into *WT* and *ASK1^−/−^* mice. After 2 mo infection with SS-1, *ASK1^−/−^* mice transplanted with *WT* bone marrow (*WT*→*ASK1^−/−^*) exhibited similar levels of inflammation, atrophy, and metaplasia to control *WT* transplanted mice (*WT*→*WT*) (Figure 3C,D). Immunoblotting of the stomach tissue from these mice showed decreased activity of JNK, p38, and downstream ATF and MK2, while similar levels of STAT1 and NF-κB activation (Figure 3E). IHC with phospho-p38 and phospho-JNK revealed that activation of these molecules was reduced in the epithelial cells, but not in the myeloid cells (Figure 3F). In the *WT*→*ASK1^−/−^* mouse stomach, the number of cyclin D1 and BrDU positive proliferating cells were decreased compared to *WT*→*WT* mice (Figure 3G,H). To analyze epithelial apoptosis after *H. pylori* infection, we performed TUNEL staining. In the oxyntic gland, we found rare TUNEL-positive cells, but no difference was seen between *WT*→*WT* and *WT*→*ASK1^−/−^* mice (Figure 3G,H). These results indicate that ASK1 in the epithelial cells is not associated with the enhanced gastric inflammation and metaplastic changes seen in systemic *ASK1^−/−^* mice, but has a promoting effect on epithelial cell proliferation.

### 3.5. ASK1 Deficiency Promotes TH1-Dependent Immune Response and Recruits Immature Gr-1+CD11b+ Cells

**Figure 4:**
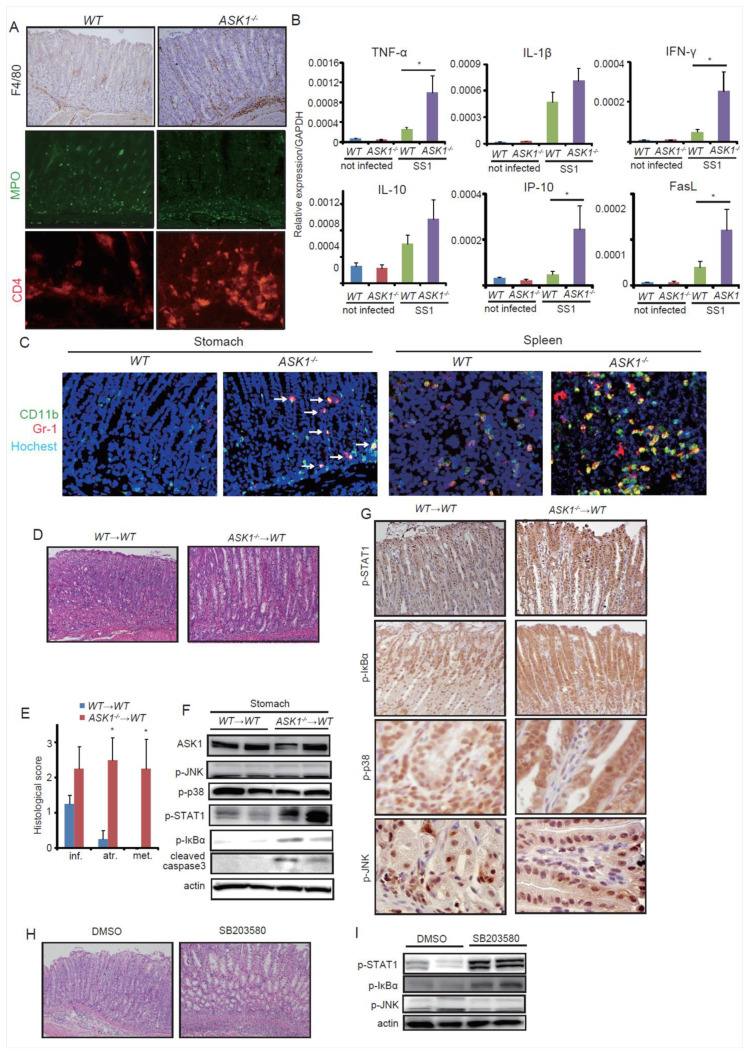
Impaired ASK1-p38 pathway in myeloid lineage promotes atrophic and metaplastic changes through NF-κB and STAT1 activation. (**A**) Immunostaining of immune cell markers in SS-1-infected *WT* and *ASK1^−/−^* mouse stomachs. Original magnification: CD4, ×200; others, ×100. (**B**) Relative mRNA expression per GAPDH in *WT* and *ASK1^−/−^* mouse stomachs with or without SS-1 infection. Data are shown as mean ± SE. * *p* < 0.05 compared to infected *WT* mice. (**C**) Immunofluorescence of CD11b (green) and Gr-1 (red) in SS-1-infected *WT* and *ASK1^−/−^* mouse stomachs and spleens. Original magnification, ×200. (**D**) H&E staining of *WT* mice transplanted with *WT* (*WT*→*WT*) or *ASK1^−/−^* (*ASK1^−/−^*→*WT*) bone marrow. Mice were sacrificed 3 mo after SS-1 infection. Original magnification, ×100. (**E**–**G**) Histological scoring (**E**), immunoblotting (**F**), and immunohistochemical staining (**G**) of SS-1-infected *WT*→*WT* and *WT*→*ASK1^−/−^* mice. Original magnification: p-p38 and p-JNK, ×400; others, ×200. Data are shown as mean ± SE. * *p* < 0.05 compared to infected *WT*→*WT* mice. (**H**,**I**) **H**&**E** staining (**H**) and immunoblotting (**I**) of *H. pylori*-infected mouse stomach treated with or without SB203580. Mice were sacrificed 3 mo after SS-1 infection. Original magnification, ×100.

To further elucidate the direct cause of dramatic metaplasic changes in *ASK1^−/−^* stomach, we assessed the inflammatory profile after *H. pylori* infection in both genotypes. To investigate which types of inflammatory cells were recruited in *H. pylori*-infected *WT* and *ASK1^−/−^* stomach, we stained with immune cell surface markers. F4/80, myeloperoxidase (MPO), and CD4 staining indicated a marked increase in the recruitment of macrophages, neutrophils, and T lymphocytes in *ASK1^−/−^* stomach (Figure 4A). When we analyzed the expression of inflammatory cytokines by real-time PCR, Th1-polarized factors TNF-ɑ, IFN-γ, and IP-10 were significantly upregulated in *H. pylori*-infected *ASK1^−/−^* stomach compared to infected *WT* stomach, while the induction of IL-1β and IL-10, which were reported as important for gastric inflammation [18,60], were not significantly different between these genotypes (Figure 4B). It has been reported that IFN-γ could induce FasL production and that FasL may play a role in the development of gastric atrophy [61,62,63]. Indeed, our data showed the significant increase of FasL expression in *ASK1^−/−^* stomach compared to *WT* stomach (Figure 4B). Moreover, *ASK1^−/−^* stomach showed the increase of Gr-1+Cd11b+ immature myeloid cells (Figure 4C), which have been reported as a critical regulator of inflammation-associated cancer [18,64,65]. Gr-1+Cd11b+ MDSCs were also increased in *ASK1^−/−^* spleen (Figure 4C), suggesting that myeloid maturation was inhibited in *ASK1^−/−^* mice and that immature MDSCs were recruited to the stomach from the spleen in response to exaggerated inflammation. These results suggest that in *ASK1^−/−^* stomach, Th1-dependent immune responses were more activated than *WT* mice, and that the recruitment of immature myeloid cells and the increased production of cytokines and apoptotic ligands may lead to the development of gastric atrophy and metaplasia.

### 3.6. Lack of ASK1 and p38 Activation in Myeloid Lineage Induced Atrophic and Metaplastic Changes through NF-κB and STAT1 Activation

To investigate the role of ASK1 in myeloid cells, we next transplanted *WT* and *ASK1^−/−^* bone marrow into *WT* mice (*WT*→*WT* and *ASK1^−/−^*→*WT*). Interestingly, *ASK1^−/−^*→*WT* mice infected with SS-1 for 2 months exhibited more severe gastric atrophy and metaplasia than *WT*→*WT* mice (Figure 4D,E). In the stomach, STAT1 and NF-κB activation were enhanced in *ASK1^−/−^*→*WT* mice, while the levels of p38 and JNK activation did not differ from *WT*→*WT* mice (Figure 4F). IHC with these molecules confirmed that STAT1 and NF-κB were activated in epithelial cells, and that p38 and JNK activation were decreased only in myeloid cells of *ASK1^−/−^*→*WT* mice (Figure 4G). These results suggest that decreased activity of p38 and JNK in *ASK1^−/−^* myeloid cells causes STAT1 and NF-κB activation in epithelial cells, followed by atrophic and metaplastic changes.

To address whether p38 or JNK is essential for the protection of *H. pylori*-induced mucosal changes, pharmacological inhibition of these molecules was performed. We found that treatment with p38 inhibitor SB203580 worsened *H. pylori*-induced atrophy and metaplasia (Figure 4H), while JNK inhibitor SP600125 treatment did not (not shown). Treatment with p38 inhibitor induced STAT1 and NF-κB activation in the stomach, as seen in *ASK1^−/−^* or *ASK1^−/−^*→*WT* mice (Figure 4I), suggesting that ASK1-p38 pathway in myeloid cells is a critical regulator of *H. pylori*-induced gastritis and metaplasia.

### 3.7. ASK1 and p38 Suppressed Macrophage Cell Death and IL-1β Secretion

**Figure 5:**
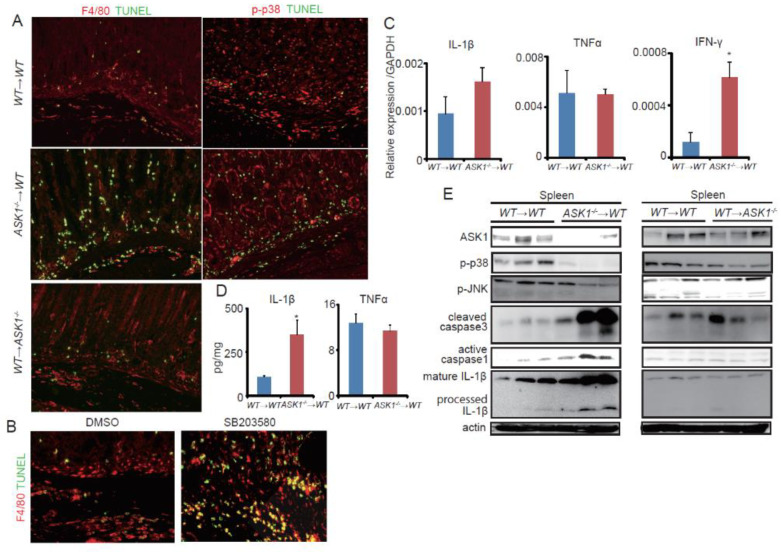
ASK1 and p38 suppressed macrophage cell death and IL-1β secretion in the stomach and spleen. (**A**) Immunofluorescent staining of SS-1-infected stomach isolated from *WT*→*WT*, *ASK1^−/−^*→*WT*, and *WT*→*ASK1^−/−^* mice. Red, F4/80 and p-p38; Green, TUNEL. Original magnification, ×200. (**B**) Immunofluorescent staining of SS-1-infected stomach treated with DMSO or SB203580. Red, F4/80; Green, TUNEL. Original magnification, ×200. (**C**) Relative mRNA expression in *WT*→*WT* and *ASK1^−/−^*→*WT* mouse spleens with SS-1 infection. Data are shown as mean ± SE. * *p* < 0.05 compared to infected *WT*→*WT* mice. (**D**) Protein levels of the indicated cytokines in *WT*→*WT* and *ASK1^−/−^*→*WT* mouse spleens measured by ELISA. Data are shown as mean ± SE. * *p* < 0.05 compared to infected *WT*→*WT* mice. (**E**) Immunoblotting of *WT*→*WT*, *ASK1^−/−^*→*WT*, and *WT*→*ASK1^−/−^* mouse spleens with SS-1 infection. Data are shown as mean ± SE. * *p* < 0.05.

As we previously reported that ASK1 protects macrophage apoptosis in the models of colitis [37], we examined macrophage apoptosis in *H. pylori*-infected gastric tissues by TUNEL staining (Figure 5A). In *H. pylori*-infected *WT*→*WT* mouse stomach, rare TUNEL-positive cells were seen in both stromal and epithelial cells. However, in *ASK1^−/−^*→*WT* mice, a greater number of TUNEL-positive cells were found in the submucosa than control groups, while *WT*→*ASK1^−/−^* mouse stomach appears similar to control group. Increased apoptosis in the stomach was confirmed by immunoblotting with cleaved caspase-3 (Figure 4F). Double staining with F4/80 showed that the majority of these apoptotic cells were F4/80-positive macrophages (Figure 5A). Indeed, CD4 or MPO-positive cells were rarely positive for TUNEL staining in both genotypes (Figure S4). Thus, *ASK1^−/−^* macrophage were more susceptible to cell death in *H. pylori*-infected stomach, as seen in the previous colitis models [37]. Although most of inflammatory cells were phospho-p38-positive in *WT*→*WT* mice, inflammatory cells in *ASK1^−/−^*→*WT* mouse stomach were mostly phospho-p38-negative (Figure 5A). These phospho-p38-negative immune cells were likely to positive for TUNEL staining (Figure 5A), and treatment with p38 inhibitor SB203580 increased apoptosis in F4/80 positive macrophages (Figure 5B). Thus, ASK1-p38 pathway likely protects macrophage cell death.

Previous papers reported that processed IL-1β was released from dead macrophage through caspase-1 dependent pathway [66]. Thus, we investigated the expression of IL-1β and other cytokines in *H. pylori*-infected mouse spleen. We found no significant differences in mRNA expression of IL-1β or TNF-ɑ between *WT*→*WT* and *ASK1^−/−^*→*WT* mouse spleens (Figure 5C). On the other hand, protein level of IL-1β was found by ELISA to be increased in *ASK1^−/−^*→*WT* spleen, but TNF-ɑ protein was not upregulated (Figure 5D). Immunoblotting of these spleens revealed that *ASK1^−/−^*→*WT* spleen exhibited lower levels of phospho-p38 and phospho-JNK with higher levels of cleaved caspase-3, active form of caspase-1, and processed or matured form of IL-1β (Figure 5E). In contrast, *WT*→*ASK1^−/−^* spleen showed similar levels of these molecules to *WT*→*WT* spleen (Figure 5E). We also found upregulation of IFN-γ in *ASK1^−/−^*→*WT* spleen as seen in *ASK1^−/−^* stomach (Figure 5C), indicating that T cells were activated in *ASK1^−/−^* spleen and stomach. Taken together, lack of ASK1 and p38 induced macrophage apoptosis in *Hp*-infected stomach and spleen, and overload of processed IL-1β and IFN-γ production appears to be a critical inducer of epithelial changes.

### 3.8. ASK1 Deficiency Induced the Expansion of Facultative Gastric Progenitor Cells

**Figure 6:**
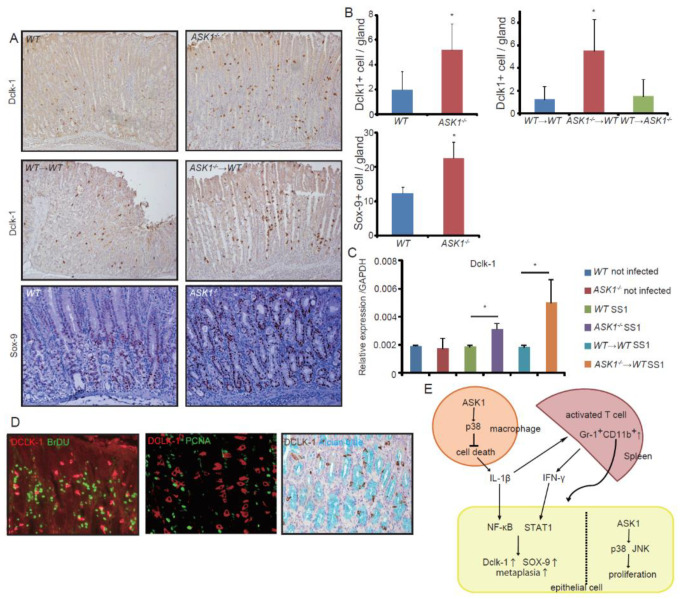
ASK1 deficiency induced the expansion of gastric facultative progenitor cells. (**A**,**B**) Dclk-1 and Sox9 staining (**A**) and the numbers of positive cells per gland in the indicated mouse stomachs. Original magnification, ×100. (**C**) Relative Dclk1 mRNA expression in the indicated mouse stomachs. (**D**) Immunohistochemical staining of the indicated markers in *ASK1^−/−^* mouse stomach infected with SS-1. Original magnification, ×200. (**E**) Schematic model of the roles of ASK1 in myeloid cells, spleen, and epithelium.

Previous studies have reported that stem/progenitor cells play a critical role in maintaining gastric mucosal homeostasis and carcinogenesis [8,10,13]. In the gastrointestine, Dclk1+ tuft cells are thought to be facultative progenitors that act as an origin of cancers in a specific condition [25,67,68,69]. Thus, we examined the distribution of Dclk1-positive cells in *WT* and *ASK1^−/−^* stomach. In *ASK1^−/−^* and *ASK1^−/−^*→*WT* stomach, the numbers of Dclk1-positive cells were significantly increased compared to *WT* and *WT*→*WT* stomach (Figure 6A,B). The expression level of *Dclk1* mRNA was also upregulated in *ASK1^−/−^* and *ASK1^−/−^*→*WT* stomach (Figure 6C). The number of cells expressing another stem/progenitor cell marker Sox9 [19] was also increased in *ASK1^−/−^* stomach compared to *WT* mice (Figure 6A,B). 

Even in the inflamed stomach, Dclk1-positive cells remain BrDU-negative, i.e., quiescent, however, those were accumulated very close to BrDU-positive or PCNA-positive isthmus stem/progenitor cells (Figure 6D). In addition, plenty of Dclk1-positive cells were observed in Alcian blue-positive, BrDU-negative metaplastic gland (Figure 6D). Therefore, largely quiescent Dclk1-positive tuft cells expand within the inflamed mucosa of *ASK1*^−/−^ mice, and may support the isthmus proliferation and development of metaplasia as a niche cell. 

In summary, we observed that the lack of ASK1 in myeloid cells dramatically affected *H. pylori*-induced epithelial changes, including atrophy, metaplasia, and the expansion of gastric progenitors through disrupted immune responses (Figure 6E). In contrast, epithelial ASK1 is important for stem/progenitor cell proliferation. ASK1 and downstream molecules have multiple functions on gastric inflammation and homeostasis.

## 4. Discussion

In this report, we showed that ASK1 deficiency promoted gastric inflammation, atrophy, and metaplasia after *H. pylori* infection. On the other hand, we demonstrated that ASK1 and downstream MAPKs are activated in gastric stem/progenitor cells and important for their proliferation. In infected *ASK1^−/−^* mice, a variety of inflammatory cells were involved and TH1-immune responses were greatly enhanced. Further, Gr1+Cd11b+ MDSCs were recruited to *ASK1^−/−^* stomach and spleen. We found that ASK1-p38 pathway in myeloid cells was important for macrophage cell death and following IL-1β secretion. These exaggerated immune responses activated NF-κB and STAT1 signaling in the epithelium, and caused dramatic expansion of facultative gastric progenitor cells.

Our bone marrow transplantation results found that epithelial ASK1 was important for isthmus progenitor proliferation, but dispensable for SPEM formation. We showed that H. pylori activates ASK1 and MAPK pathway in part through production of reactive oxygen species (ROS) in gastric epithelial cells [70]. Since ROS production by *H. pylori* appears to be CagA-dependent as shown in previous studies [71,72], CagA translocation may contribute to ASK1 activation in isthmus progenitor cells. Nevertheless, it remains undetermined how and where metaplastic cells come from after *H. pylori* infection. In mouse models, several reports have shown that SPEM may be transdifferentiated from mature chief cells [5,73], while we and others reported that metaplasia arises from isthmus stem/progenitor cells [7,10,52,74,75,76]. Our current data suggest that while the strong MAPK activation in the isthmus and pit cells regulates their proliferation, development of SPEM, in which MAPK activity is absent, may not be directly affected by MAPK activation, but depends more on the degree of inflammation. 

For stem cell maintenance, Wnt signaling activation is thought to be most critical. In the mouse corpus, Wnt5a-dependent noncanonical Wnt signaling appears to be involved in stem cell function [10,14]. While other pathways such as gastrin, acetylcholine, R-spondin, BMP signaling likely contribute to gastric stem cell niche [11,77,78,79,80,81,82,83], accumulating evidence highlighted the importance of MAPK pathway in gut stem cells. For example, it has been reported that JNK is one of Wnt activators in the intestine and thus promotes epithelial proliferation and carcinogenesis [84]. Gastrointestinal organoid culture often requires pharmacological inhibition of p38MAPK [85], and p38MAPK activation is reported to be involved in gastrointestinal stem cell aging [86]. Interaction between ASK1 and Notch signaling [87], which is known to expand gastric stem cell differentiation [88], was also reported. Thus, ASK1 and other MAPK pathways could be functionally important in gastric stem/progenitor cells. Further analysis would be required for exploring the detailed mechanism in which ASK1-MAPK pathway regulates stem cell functions.

We previously reported that ASK1–JNK pathway plays a critical role on gastric carcinogenesis by controlling epithelial cell proliferation [35,89]. In these studies, we used MNU-induced chemically induced gastric tumor model. Given that most of MNU-induced tumors arise from the mouse antrum or pylorus, the mechanism of MNU-tumor development must be different from the way of corpus metaplasia or tumor. Indeed, corpus glands are in many aspects distinct from antral glands, with distinguished gland structure containing different stem/progenitor cells as well as different responses to external stimulants including carcinogens and gastrin hormones [12,13,33,50,74,90,91]. Nevertheless, MAPK activation can be found both in the antrum and corpus, in particular in the area between the isthmus and the pit cells. Thus, MAPK activation in pit cells and isthmus progenitors is important for cell proliferation in both regions, and that MNU-induced tumor, whose development is suppressed by ASK1 deficiency, would be most likely derived from these cell zone. 

Our present study revealed that ASK1 deficiency in myeloid lineage caused macrophage apoptosis and IL-1β secretion, which is consistent with our previous study of colitis model [37]. IL-1 signaling has been reported to play an important role on gastric carcinogenesis [18,19,20,27,58]. While it remains possible that IL-1β can directly activate epithelial cell population, our current data and past studies suggest that IL-1β is a key mediator for broad immune reaction. Indeed, we demonstrated that innate immune disorder induced by IL-1β release triggered adaptive immune responses including IFN-γ and FasL production, and also initiated the recruitment of Gr-1+CD11b+ MDSCs. Several previous papers have shown that MDSCs could be engaged by abnormal cytokine production such as IL-1β, TNF-ɑ, and IFN-γ [18,92,93,94]. However, it remains unclear whether MDSCs are induced by higher levels of IL-1β or IFN-γ in *ASK1^−/−^* mice, or ASK1 deficiency causes the impairment of myeloid cell maturation. Future investigation would be needed concerning the relationship between ASK1 and myeloid cell maturation.

Stem cell niche contributes to stem cell maintenance as a source of growth factors and cytokines. Abnormal immune cells and excessive levels of cytokines around gastric stem cells could change their survival and functions. Thus, we here raise the conclusion that disrupted immune responses in *ASK1^−/−^* mice including innate immunity, adaptive immunity, and MDSCs, alter the gastric stem cell niche, which eventually cause progenitor cell expansion and metaplastic changes.

## Supplementary Materials

The following are available online at https://www.mdpi.com/2076-2607/8/12/1995/s1, Supplementary Figure S1. PMSS-1 infection experiments. (**A**) Representative H&E staining of *WT* and *ASK1^−/−^* mouse stomach with *H. pylori* PMSS-1 infection. Original magnification, ×100. (**B**) Histological scoring of PMSS-1-infected *WT* and *ASK1^−/−^* mouse stomach. Inf., inflammation; atr., atrophy; met., metaplasia; hyp., hyperplasia. Data are shown as mean ± SD. * *p* < 0.05 compared to infected *WT* mice. (**C**) Colony-forming units of *H. pylori* isolated from the infected *WT* and *ASK1^−/−^* mouse stomach. Supplementary Figure S2. ASK1, not ASK2, is responsible for downstream MAPK activation in the stomach. (**A**) Immunoblotting of *WT* and *ASK2^−/−^* mouse stomach and spleen infected with SS-1. (**B**) Total p38 staining in SS-1-infected *WT* and *ASK1^−/−^* stomach. Original magnification, ×200. Supplementary Figure S3. ASK1 deficiency induced NF-κB and STAT1 activation in the PMSS-1 infected stomach. Immunoblotting of PMSS-1-infected *WT* and *ASK1^−/−^* mouse stomachs. Supplementary Figure S4. ASK1 deficiency did not promote apoptosis in T cells and neutrophils. Immunofluorescent staining combined with TUNEL assay in SS-1-infected *WT* and *ASK1^−/−^* stomach and spleen. Original magnification, ×200.
