# Supplementary material for: Dysregulated Immune Responses by ASK1 Deficiency Alter Epithelial Progenitor Cell Fate and Accelerate Metaplasia Development during H. pylori Infection
Source: Microorganisms. 2020 Dec 14;8(12):1995. doi: 10.3390/microorganisms8121995 (PMC7765114; doi:10.3390/microorganisms8121995)
Supplement: Supplementary file 1 [file microorganisms-08-01995-s001.pdf]

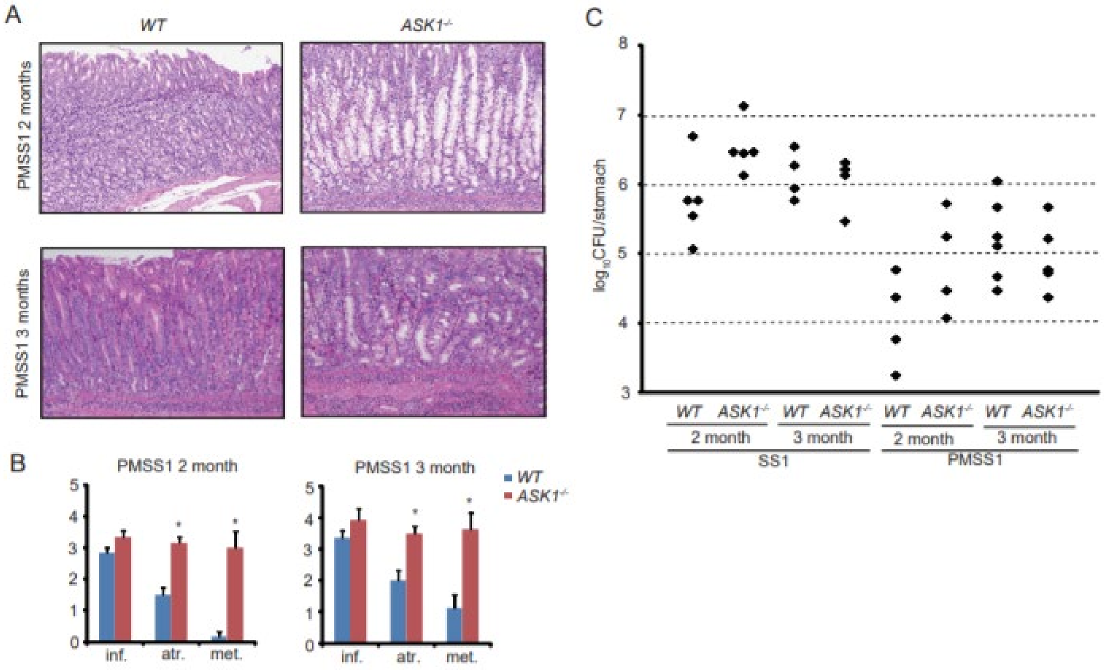

Supplementary Figure S1. PMSS-1 infection experiments. (**A**) Representative H&E staining of *WT* and *ASK1*$^{-/-}$ mouse stomach with *H. pylori* PMSS-1 infection. Original magnification, ×100. (**B**) Histological scoring of PMSS-1-infected *WT* and *ASK1*$^{-/-}$ mouse stomach. Inf., inflammation; atr., atrophy; met., metaplasia; hyp., hyperplasia. Data are shown as mean ± SD. **P* < 0.05 compared to infected *WT* mice. (**C**) Colony-forming units of *H. pylori* isolated from the infected *WT* and *ASK1*$^{-/-}$ mouse stomach.

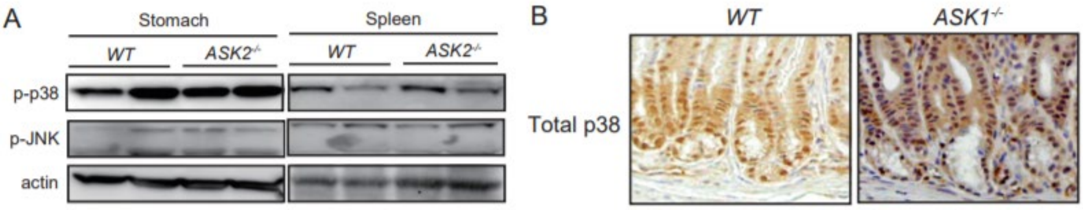

Supplementary Figure S2. ASK1, not ASK2, is responsible for downstream MAPK activation in the stomach. (**A**) Immunoblotting of *WT* and *ASK2*$^{-/-}$ mouse stomach and spleen infected with SS-1. (**B**) Total p38 staining in SS-1-infected *WT* and *ASK1*$^{-/-}$ stomach. Original magnification, ×200.

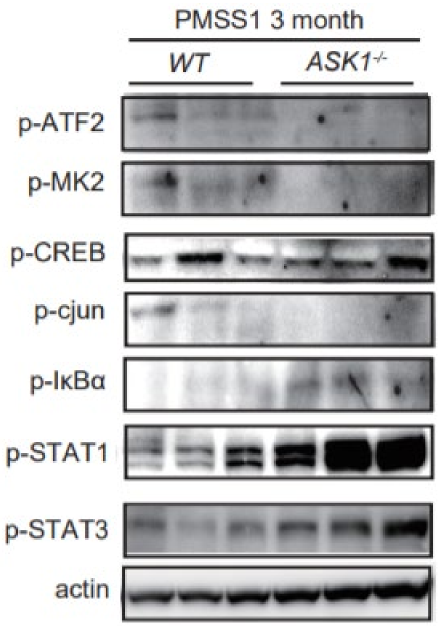

Supplementary Figure S3. ASK1 deficiency induced NF-κB and STAT1 activation in the PMSS-1 infected stomach. Immunoblotting of PMSS-1-infected *WT* and *ASK1*$^{-/-}$ mouse stomachs.

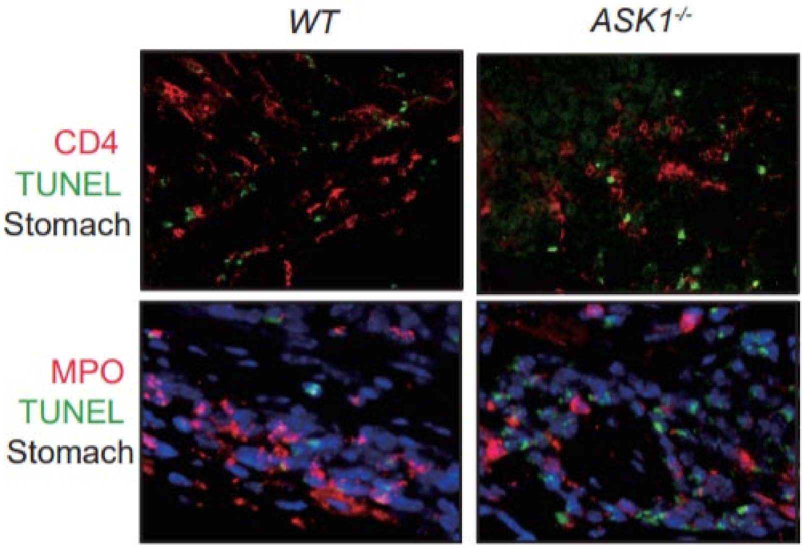

Supplementary Figure S4. ASK1 deficiency did not promote apoptosis in T cells and neutrophils. Immunofluorescent staining combined with TUNEL assay in SS-1-infected *WT* and *ASK1*$^{-/-}$ stomach and spleen. Original magnification, ×200.
